# A Rare Complication of Asthma: Retropharyngeal Emphysema, Subcutaneous Emphysema, and Pneumomediastinum

**DOI:** 10.7759/cureus.10524

**Published:** 2020-09-18

**Authors:** Ahsum Khan, Asiya Tafader, Rafae Shaikh, Jason Jacob

**Affiliations:** 1 Internal Medicine, University of Connecticut School of Medicine, Farmington, USA; 2 Internal Medicine, UConn Health, Farmington, USA; 3 Medicine, Hartford Hospital, Hartford, USA

**Keywords:** pneumomediastinum, subcutaneous emphysema, retropharyngeal emphysema, asthma

## Abstract

Spontaneous pneumomediastinum (SPM) is a relatively uncommon occurrence. Although unlikely, asthma exacerbations can produce enough barotrauma to produce this complication. In cases of SPM, the gas has the opportunity to track between fascial planes, making its way to subcutaneous tissues, usually of the neck and chest, resulting in subcutaneous emphysema (SE). In anomalous situations, this gas can track its way into the retropharyngeal space. This presentation is usually self-limiting, requiring supportive therapy. Severe cases can lead to airway compromise warranting invasive supportive airway maneuvers. Retropharyngeal emphysema, SE, and pneumomediastinum have rarely been described together in the literature. This case provides awareness of these three complications of asthma, while highlighting the need for deliberate chest imaging, including radiograph and non-contrast CT, in patients with severe asthma exacerbations.

## Introduction

Spontaneous pneumomediastinum (SPM) describes the presence of extraluminal gas within the mediastinum, not preceded by surgery, thoracic trauma, or any medical procedure. Associated complications with pneumomediastinum can include subcutaneous emphysema (SE) that describes the presence of gas within the subcutaneous tissues. The gas produced internally, during a pneumomediastinum, can track from the mediastinum to the subcutaneous tissues, usually of the neck and chest. This gas may also make its way into the retropharyngeal fascia, producing a rare clinical picture of retropharyngeal emphysema (RPE) [1-5].

In this report, we describe the case of a 23-year-old female with a past medical history of intermittent asthma, initially presenting for an acute asthma exacerbation in the setting of rhinovirus infection, who consequently developed left neck swelling with pain, decreased range of motion, and odynophagia, found to have SE with retropharyngeal air on imaging.

A literature review revealed only a handful of cases reporting the occurrence of SE and SPM in the setting of various respiratory viral infections and only one pediatric case that describes these complications in the setting of rhinovirus infection. No adult cases have been reported [1-4]. Furthermore, the synchronous presentation of RPE, SPM, and SE has rarely been reported in the setting of asthma.

## Case presentation

**Figure 1:**
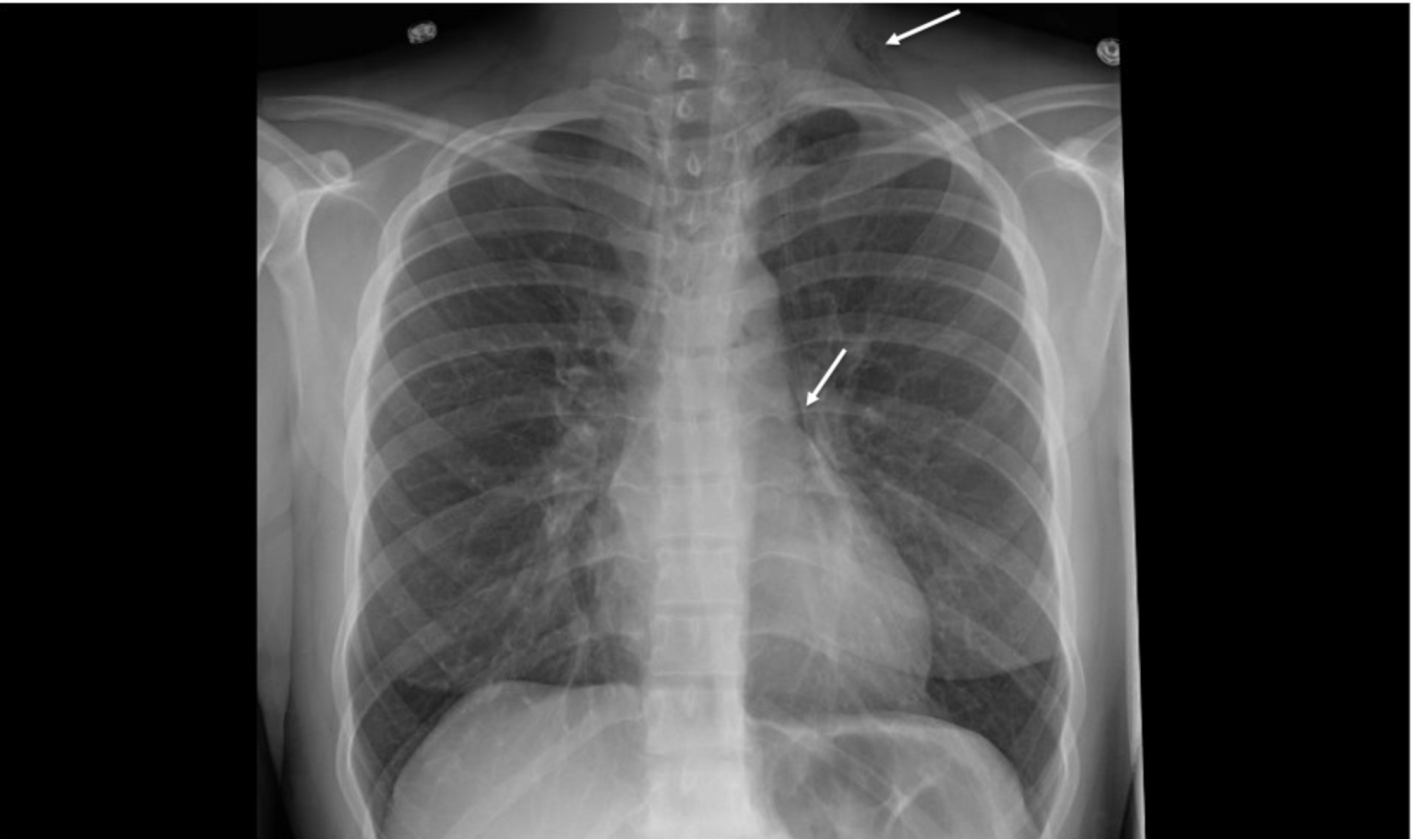
Plain chest radiograph demonstrating pneumomediastinum (linear lucency outlining left heart border - lower white arrow) and subcutaneous emphysema (lucency visualized in left lower neck - upper white arrow)

A 23-year-old white female with a past medical history of intermittent asthma controlled on albuterol, presented to the emergency department (ED) at an outside hospital with a four-day history of worsening dry cough, shortness of breath, and wheezing. The patient had never been hospitalized or intubated due to her asthma, but had presented to the ED twice for her symptoms. Both times she was discharged from the ED, after receiving nebulizer and oral prednisone treatment. She noted this exacerbation was out of proportion compared to her usual asthma symptoms, which were well controlled with as needed albuterol. During her admission, she received DuoNeb and oral prednisone treatment with mild improvement in her symptom burden. However, she soon developed a productive cough with yellow sputum, a sharp pain in her left lower neck, chest, limited neck range of motion, and an associated sensation of bubbles she could hear and feel in the affected area. A chest radiograph was performed that revealed SE in her left lower neck, as well as pneumomediastinum around her left and right heart borders. Given her imaging findings, she was transferred to our hospital for further investigation. The radiograph is shown in Figure 1.

**Figure 2:**
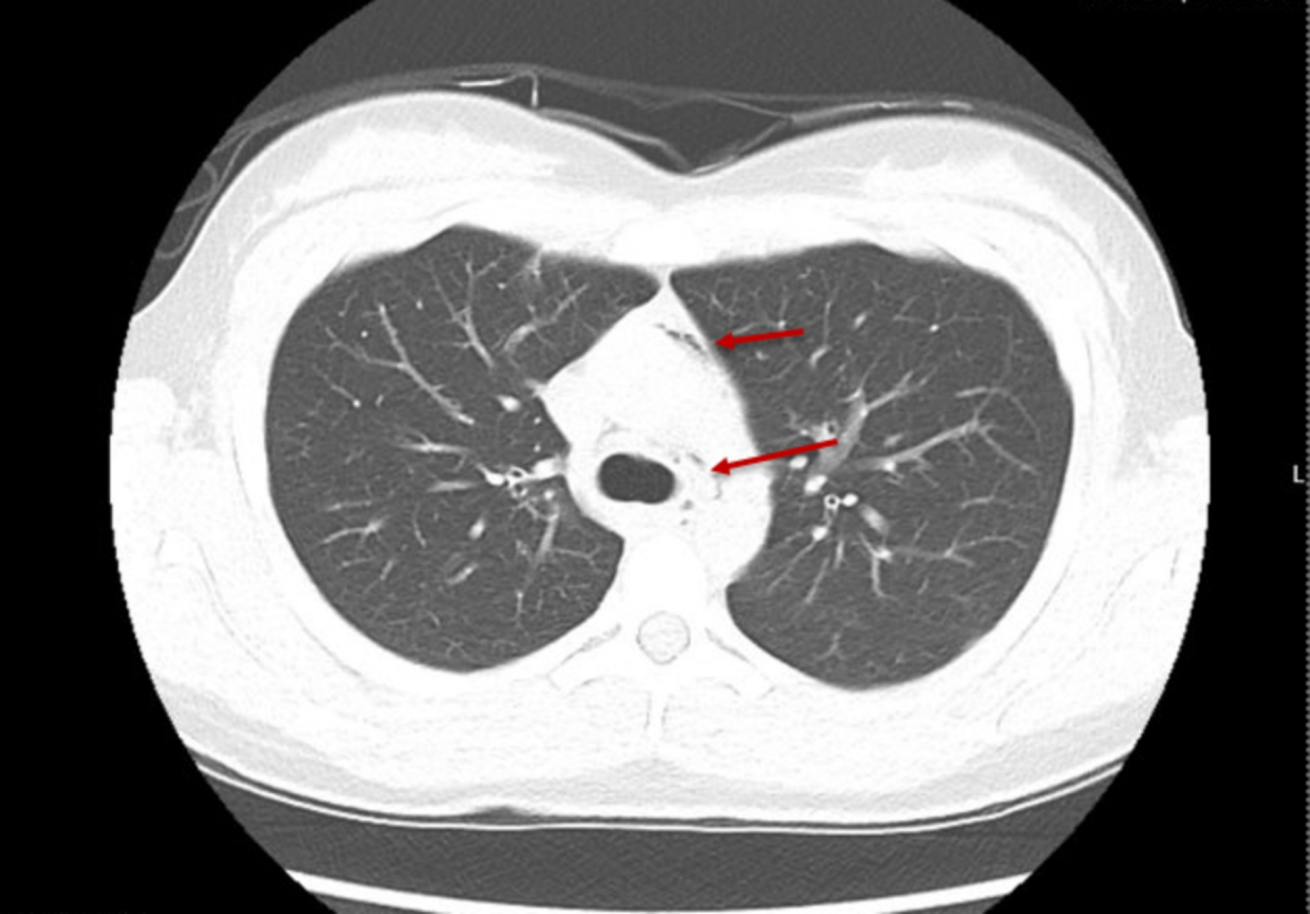
CT of the chest demonstrating the presence of air within the mediastinum, surrounding the pulmonary trunk (upper red arrow) and around the trachea (lower red arrow)

**Figure 3:**
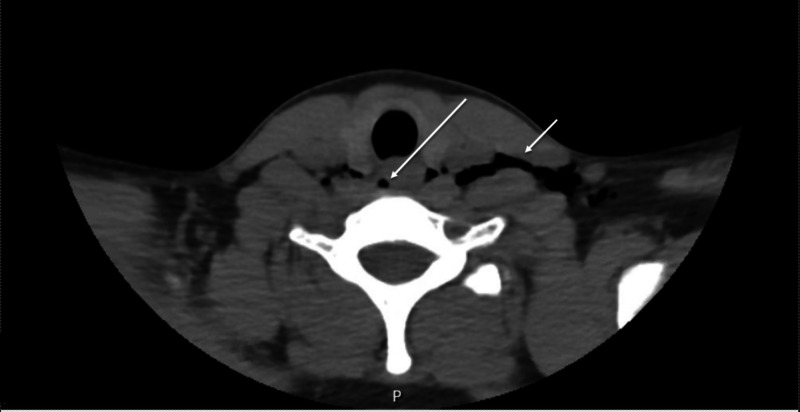
CT of the neck demonstrating subcutaneous emphysema (hypodense gas denoted by the right white arrow) and retropharyngeal emphysema (hypodense gas denoted by the left white arrow)

Upon arrival, she was hemodynamically stable with vital signs significant for tachycardia to 118 beats per minute, tachypnea to 22 breaths per minute and requiring 2 L of oxygen via nasal cannula to maintain 95% oxygen saturation. On initial evaluation, the patient was not in any respiratory distress, but noted persistent sharp left neck and chest pain. She also complained of odynophagia. She denied any fever, chills, headache, lightheadedness, dizziness, abdominal pain, or diarrhea. On physical exam, there was palpable crepitus present in her left lower neck, as well as a positive Hamman's sign on cardiac auscultation. A fluoroscopic esophagogram was obtained that revealed no signs or evidence of leakage of contrast out of the esophagus and no evidence of obstruction. A CT neck and chest was also obtained that revealed SPM, RPE, and SE, within her left lower neck (Figures 2, 3).

Over two days, the patient was given scheduled albuterol, a prednisone taper, and baclofen (for her neck pain) and demonstrated improvement. She was eventually weaned off her oxygen to satisfactory ambulation oxygenation. Her cough, shortness of breath, and neck pain significantly improved as well. A outpatient pulmonary medicine follow-up was scheduled in two weeks and she was instructed to continue using her fluticasone inhaler, her albuterol four times per day, finish her prednisone taper over the course of one week, and finish her baclofen over the course of five days.

## Discussion

This case highlights an unusual complication of an asthma exacerbation. A presentation of SPM, SE, and RPE were most likely related to the sudden rise in intra-alveolar pressure with severe coughing or deep inspiration related to her obstructive lung disease. The sudden rise in intra-alveolar pressure ultimately leads to rupture, creating a path for extraluminal gas to possibly enter mediastinal, subcutaneous, retropharyngeal, retroperitoneal, and even epidural areas [6,7]. In our patient, air tracked from ruptured alveoli within the bronchovascular fascia back towards the hilum of the lung into mediastinal, subcutaneous structures in the neck and retropharyngeal areas (most prominent at the level of thyroid).

SPM may originate from a variety of regions, including the esophagus, lungs, trachea, or peritoneal cavity. Factors such as chest/barotrauma, neck/retroperitoneal surgery, esophageal perforation, tracheobronchial perforation, infection, interstitial lung disease, and cocaine use have all been implicated in producing pneumomediastinum [1]. Asthma has also been rarely implicated in these cases, due to the forced inhalation/severe coughing seen in asthma exacerbations. Overall, SPM is a rare entity and has a reported incidence of 1 in 45,000, with a male preponderance (76%) [1-3].

In regard to clinical picture, pneumomediastinum presents with the triad of acute substernal chest pain (80%-91%), severe dyspnea (30%), and neck pain (20%). A positive Hamman's sign (40%-80% of cases), in which a crunching noise is heard synchronously with each heartbeat on auscultation (best heard in the left lateral decubitus position), can also be seen. In the most severe cases, cyanosis, pulsus paradoxus, and hemodynamic impairment can be seen [6,7].

Causes of SE can be divided into three groups: gas produced internally (pneumothorax, pneumomediastinum, fistula, esophageal perforation), externally (penetrating trauma or post-surgical intervention), or de novo (necrotizing fasciitis). Palpable crepitus is seen on exam with SE. In severe cases, a buildup of a large amount of air can occur resulting in the compression of nearby structures resulting in worsening hypoxia and hypercarbia [6,7].

Complications in SPM can include RPE and secondary pneumothorax. RPE is a complication most often reported in dental procedures or traumatic aerodigestive tract injury [6]. In our patient, the RPE was present in the context of an associated pneumomediastinum, likely due to air tracking. Clinically, these patients can present with dysphagia and odynophagia, which is an important clue [8]. Subpleural emphysema is also a serious complication in SPM that can develop if the gas from the ruptured alveoli tracks to the lung pleura, leading to the formation of sub pleural bullae. These bullae have the potential to rupture leading to a secondary pneumothorax [1,2,8].

Diagnosis of SPM and SE should first be confirmed via chest radiograph. On radiograph, lucencies (gas) in subcutaneous tissues as well as gas dissecting/outlining the mediastinal structures can be visualized. If radiograph is inconclusive or further investigation is warranted, a CT exam can be ordered, which can provide further evidence of extraluminal gas, as well as more precise information of where the gas may have tracked. This is particularly true, when investigating for RPE, which can only be confirmed via CT. If an esophageal rupture is in the differential, a fluoroscopic esophagogram can be obtained, as was the case in our patient. If contrast is seen freely flowing through the esophagus without obstruction or diffusing out of the esophagus, rupture and obstruction are both much less likely [4-6].

Management of this triad of presentation is usually conservative. Spontaneous resolution is usually achieved in most patients after two to seven days, in the context of good asthma control (bronchodilators, steroids, oxygen therapy if needed) and supportive therapy [8,9]. In our patient, she was eventually weaned off her oxygen requirement, after consistent DuoNeb and oral prednisone treatments. Baclofen proved to be helpful for her neck and throat pain, while improving her range of motion. She was discharged with close follow-up.

## Conclusions

This report describes a case of synchronous SPM, SE, and RPE as a complication of an asthma exacerbation. Air tracking from ruptured alveoli into extrapulmonary tissues is an uncommon yet important complication to keep in mind in these patients. Although these complications usually spontaneously resolve, in the context of supportive therapy and good asthma control, there is an increased risk of recurrence in these patients (5%-10%). There is also the risk of spontaneous primary pneumothorax that occurs due to air tracking into subpleural areas, leading to subpleural blebs that are at risk of rupture. A high index of suspicion is warranted in asthmatic patients who present with chest, neck, and throat pain, along with intractable dyspnea with no improvement with medical therapy.

## References

[1] Macia I, Moya J, Ramos R (2007). Spontaneous pneumomediastinum: 41 cases. Eur J Cardiothorac Surg.

[2] Cho DY, Aaron GP, Shepard KG (2016). Spontaneous retropharyngeal and mediastinal emphysema. Clin Exp Otorhinolaryngol.

[3] Curci JJ, Horman MJ (1976). Boerhaave's syndrome: the importance of early diagnosis and treatment. Ann Surg.

[4] Paquette M, Terezhalmy GT, Moore WS (2002). Subcutaneous emphysema. Quintessence Int.

[5] López-Rivera F, Rivera XC, González Monroig HA, Puebla JG (2018). Pneumomediastinum and pneumothorax associated with herpes simplex virus (HSV) pneumonia. Am J Case Rep.

[6] Smith JL II, Hsu JM (2004). Spontaneous pneumomediastinum presenting with retropharyngeal emphysema. Am J Otolaryngol.

[7] Tortajada-Girbés M, Moreno-Prat M, Ainsa-Laguna D, Mas S (2016). Spontaneous pneumomediastinum and subcutaneous emphysema as a complication of asthma in children: case report and literature review. Ther Adv Respir Dis.

[8] Zhang XY, Zhang WX, Sheng AQ, Zhang HL, Li CC (2013). Diagnosis and prognosis of spontaneous pneumomediastinum in eighteen children. [Article in Chinese]. Zhonghua Er Ke Za Zhi.

[9] Alexiou K, Sakellaridis T, Sikalias N, Karanikas I, Economou N, Antsaklis G (2009). Subcutaneous emphysema, pneumomediastinum and pneumoperitoneum after unsuccessful ERCP: a case report. Cases J.

